# Evolution of invasive meningococcal disease epidemiology in Europe, 2008 to 2017

**DOI:** 10.2807/1560-7917.ES.2022.27.3.2002075

**Published:** 2022-01-20

**Authors:** Charles Nuttens, Jamie Findlow, Paul Balmer, David L Swerdlow, Myint Tin Tin Htar

**Affiliations:** 1 Vaccine Medical Development, Scientific and Clinical Affairs, Pfizer, Paris, France; 2 Vaccine Medical Development, Scientific and Clinical Affairs, Pfizer Ltd, Tadworth, United Kingdom; 3 Vaccine Medical Development, Scientific and Clinical Affairs, Pfizer Inc, Collegeville, PA, United States

**Keywords:** invasive meningococcal disease, epidemiology, evolution, Europe, *Neisseria meningitidis*, serogroup, adolescents, elderly

## Abstract

**Background:**

Invasive meningococcal disease (IMD) epidemiology has fluctuated over the past 25 years and varies among serogroups, age groups and geographical locations.

**Aim:**

This study analysed the evolution of European IMD epidemiology from 2008 to 2017 to identify trends.

**Methods:**

Reported number of IMD cases and associated incidence were extracted from the European Centre for Disease Prevention and Control Surveillance Atlas for Infectious Diseases for individual European countries. Epidemiology and its evolution were analysed by serogroup and age group.

**Results:**

Overall IMD incidence decreased by 34.4% between 2008 and 2017. Serogroup B remained predominant in 2017; despite a 56.1% decrease over the 10-year period, the rate of decrease has slowed in recent years and varies by age group. Serogroup C was the second most prevalent serogroup until 2016. Its incidence decreased among individuals aged 1–24 years, the main population targeted by MenC vaccination campaigns, but increases have occurred in other age groups. Incidences of serogroups W and Y were low but increased by > 500% and > 130% (to 0.10 and 0.07/100,000) respectively, from 2008 to 2017. Considering all serogroups, a marked modification of the evolution trends by age group has occurred, with increases in incidence mainly affecting older age groups.

**Conclusion:**

Although the overall IMD incidence decreased in Europe between 2008 and 2017, increases were observed for serogroups W and Y, and in the older population when considering all serogroups. It may be necessary to adapt current vaccination strategies to reflect epidemiological changes and their likely future evolution.

## Introduction

Invasive meningococcal disease (IMD) is a life-threatening illness caused by the pathogen *Neisseria meningitidis* [1]. Disease can progress from mild, non-specific illness to severe disease within hours [2], including death in ca 10% of cases [3,4]. Survivors may experience serious lifelong sequelae [5]. Meningococci are classified into 12 serogroups based on their capsular polysaccharide [6]. Collectively, serogroups A, B, C, W and Y have historically caused the majority of disease worldwide, with serogroup distribution varying across global regions.

In Europe, serogroups B, C, W and Y are responsible for more than 95% of IMD cases, with serogroup B generally predominating since at least the 1960s [4,7]. Serogroup C became an important concern when it emerged rapidly in the late 1990s but its incidence decreased substantially following the introduction of immunisation programmes in many European countries [8,9]. The incidences of IMD caused by serogroups W and Y have historically been lower in Europe compared with serogroups B and C, despite an outbreak caused by serogroup W in several European countries in 2000 and 2001 associated with the Hajj pilgrimage [4,7,10]. More recently, a hypervirulent serogroup W strain belonging to clonal complex 11 (cc11) has been responsible for noticeable increases in IMD cases in various European countries [11-14]. Clusters of IMD cases and outbreaks also occur periodically, often necessitating reactive outbreak control strategies [15,16].

The incidence of IMD is highest in infants, followed by young children; however, a secondary peak may occur among adolescents and young adults [4]. Vaccination strategies for IMD in Europe have therefore frequently focused on infants and adolescents [17]. Vaccination programmes using capsular polysaccharide-based serogroup C conjugate (MenC) vaccine, beginning in 1999, have notably impacted the epidemiology of IMD in Europe [8,18], and the more recent implementations of vaccination with serogroup A, C, W and Y conjugate (MenACWY) vaccine will probably have similar broad-ranging effects. The MenACWY vaccine programmes often include adolescents, sometimes exclusively [19-23]; this strategy can provide both direct protection to adolescents as well as indirect protection to other unvaccinated age groups such as young children and older adults, by reducing carriage and onward transmission [24]. Development of vaccines based on serogroup B (MenB) subcapsular proteins have similarly enabled the introduction of infant MenB vaccine programmes in some European countries, and confirmation of resulting vaccine effectiveness is beginning to emerge [17,25].

Understanding epidemiological evolution of a given disease is essential for optimisation of public health interventions. Prevention strategies for IMD require a detailed understanding of current IMD incidence and trends and, despite the unpredictability of disease epidemiology, should also incorporate likely future changes [4]. We undertook a study to describe the evolution of European IMD epidemiology between 2008 and 2017 to characterise recognised epidemiological trends and identify potential new ones.

## Methods

### Data sources

We extracted IMD surveillance data from 2008 to 2017 (the most recent 10 years for which data were available when the analysis was initiated) from the European Centre for Disease Prevention and Control Surveillance Atlas for Infectious Diseases as collected through The European Surveillance System (TESSy) [26]. The methodology for this data collection is described elsewhere [27]. Countries belonging to the European Economic Area as of 2017 with a population ≥ 1 million were included in the analyses and consisted of Austria, Belgium, Bulgaria, Croatia, Czechia, Denmark, Estonia, Finland, France, Germany, Greece, Hungary, Ireland, Italy, Latvia, Lithuania, the Netherlands, Norway, Poland, Portugal, Romania, Slovakia, Slovenia, Spain, Sweden and the United Kingdom (UK) (26 countries total; hereafter referred to as Europe) [28].

### Data calculation

We used country-specific incidence (overall, by serogroup, by age group, and by serogroup and age group) and number of cases (overall and by serogroup) as extracted from the Surveillance Atlas. Country-specific numbers of cases by age group, and by serogroup and age group, were calculated using the age group-specific proportion of cases occurring overall or in each serogroup. Incidence and number of cases for Europe were calculated using the number of cases for each individual country and their population estimates for the corresponding year (overall or by age group) from Eurostat [28]. If a country was missing data for a given year in the Surveillance Atlas, we omitted the population of that country when calculating the total population for that year.

Relative evolutions of incidence over time were calculated to provide meaningful comparisons and were expressed as percentage increases/decreases or fold changes. We calculated fold changes by dividing the incidence from a given year by that for the corresponding reference year. A quotient of 1 represented no relative change; quotients > 1 or < 1 indicated relative increases or decreases, respectively. Calculations were performed using exact incidence values and rounded subsequently. 

### Statistical analyses

We performed linear trend characterisation by applying a segmented linear regression model using the least-squares method. Data smoothing was performed by applying a locally weighted scatterplot smoothing (LOWESS) using a smoothing coefficient (i.e. smoother span argument; f) of 0.75. We used R 3.5.0 (R Foundation for Statistical Computing; Vienna, Austria) to perform statistical analyses and generate figures [29].

### Ethical Statement

Because publicly available surveillance data were used, ethical approval for conduct of this study was not required.

## Results

### Overview of invasive meningococcal disease epidemiology

The overall IMD incidence in Europe declined from 0.95 per 100,000 (4,744 cases) in 2008 to 0.62 per 100,000 (3,212 cases) in 2017, representing a reduction of 34.4% over the 10-year period (Table). Incidence varied considerably among individual countries, ranging from 0.27 to 3.41 per 100,000 (median: 0.79/100,000) in 2008 and from 0.10 to 2.39 per 100,000 (median: 0.48/100,000) in 2017.

**Table:** Incidence of invasive meningococcal disease, serogroup distribution and evolution in Europe, 2008 and 2017 (n = 7,956)

	2008			2017			Evolution 2008–2017	
	Incidence per 100,000		Serogroup distribution (cases), %	Incidence per 100,000		Serogroup distribution (cases), %	Incidence per 100,000 in Europe^a^	
	Europe^a^	Median (range)		Europe^a^	Median (range)		Absolute difference	Relative difference, %
All cases	0.95	0.79 (0.27–3.41)	NA	0.62	0.48 (0.10–2.39)	NA	−0.33	−34.4
Serogroup B	0.69	0.59 (0.07–3.23)	71.5	0.30	0.30 (0.05–1.16)	48.0	−0.39	−56.1
Serogroup C	0.14	0.12 (0.00–0.37)	14.4	0.10	0.07 (0.01–0.59)	15.3	−0.04	−30.4
Serogroup W	0.02	0.01 (0.00–0.12)	1.7	0.10	0.02 (0.00–0.47)	16.0	0.08	517.0
Serogroup Y	0.03	0.02 (0.00–0.13)	3.0	0.07	0.04 (0.00–0.17)	10.9	0.04	137.1
Other^b^/non-groupable	0.09	0.06 (0.00–0.44)	9.5	0.06	0.04 (0.00–1.12)	9.8	−0.03	−32.8

NA: not applicable.

^a^ 26 countries within the European Economic Area and with ≥ 1 million population are included in the analysis.

^b^ Includes serogroups A, X and E.

Comparing incidence for individual serogroups in 2017 and change since 2008 highlights important disparities (Table; Supplementary Figure S1 shows the variability of incidence between countries for each serogroup). Incidence of serogroup B IMD remained the highest in 2008 and 2017 but decreased (0.69/100,000 in 2008 to 0.30/100,000 in 2017; 56.1% reduction) concurrently with increases of serogroups W (0.02/100,000 to 0.10/100,000; 517.0% increase) and Y (0.03/100,000 to 0.07/100,000; 137.1% increase). These changes led to evolution of the serogroup distribution, with serogroup B decreasing from 71.5% of all IMD cases in 2008 to 48.0% in 2017, whereas serogroups W and Y increased from, respectively, 1.7% and 3.0% in 2008 to 16.0% and 10.9% in 2017 (Table). Incidence of serogroup C IMD decreased by 30.4% (0.14/100,000 in 2008 to 0.10/100,000 in 2017) during the study period and this serogroup represented 15.3% of all IMD cases in 2017.

### Serogroup B

**Figure 1:**
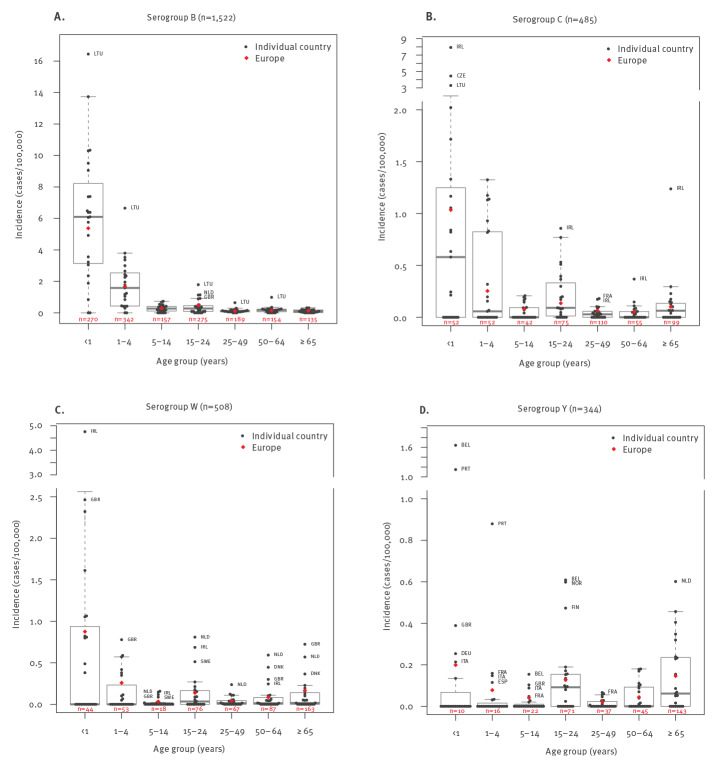
Incidence of invasive meningococcal disease caused by serogroups B, C, W and Y, by age group for individual countries and Europe, 2017 (n = 2,859)

Incidence of serogroup B IMD in Europe was 0.30 per 100,000 across all ages in 2017 (Table); however, rates varied across age groups and were highest in infants (5.37/100,000) and children aged 1–4 years (1.67/100,000). There were 612 serogroup B cases in these groups, accounting for 40.2% of the 1,522 total serogroup B IMD cases in 2017 (Figure 1). In adolescents and young adults (15–24 years-old), there were 275 cases (18.1%), with a corresponding incidence of 0.50 per 100,000. A limited number of cases (n = 135–189; 8.9–12.4%), associated with low corresponding incidence, occurred in older age groups. Incidence varied substantially among countries, ranging from 0 to 16.44 per 100,000 in infants and 0 to 6.65 per 100,000 in children aged 1–4 years. 

BEL: Belgium; CZE: Czechia; DEU: Germany; DNK: Denmark; ESP: Spain; FIN: Finland; FRA: France; GBR: UK; IMD: invasive meningococcal disease; IRL: Ireland; ITA: Italy; LTU: Lithuania; NLD: the Netherlands; NOR: Norway; PRT: Portugal; SWE: Sweden.

Data are for individual countries (black circles) and Europe (26 countries included; red diamonds). The number of IMD cases in Europe is indicated in red for each age group. Boxes indicate 25th, 50th and 75th percentiles of incidence rates for each group; whiskers indicate lowest and highest data point excluding any outliers. An incidence of 0 in specific age groups is likely to be related to the population sizes of the individual countries.

**Figure 2:**
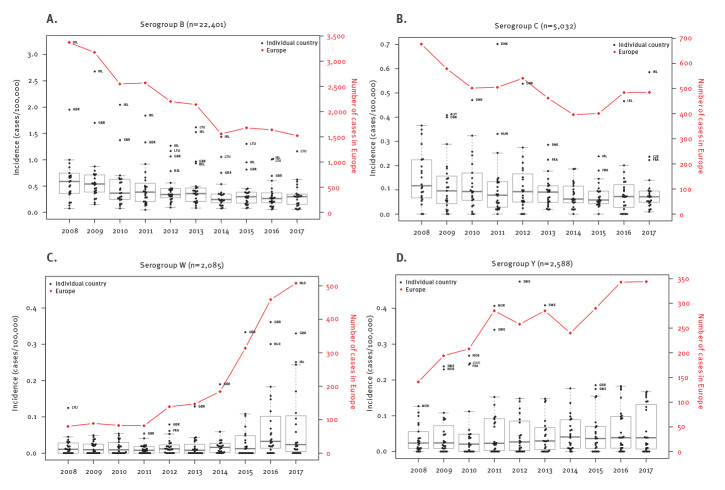
Evolution of the incidence of invasive meningococcal disease caused by serogroups B, C, W and Y for individual countries, and number of cases for Europe, 2008–2017 (n =  32,106)

AUT:  Austria; BEL:  Belgium; CZE:  Czechia; DNK:  Denmark; FIN:  Finland; FRA:  France; GBR:  UK; HUN:  Hungary; IRL:  Ireland; LTU:  Lithuania; NLD:  the Netherlands; NOR:  Norway; SWE:  Sweden.

Although serogroup B incidence in Europe decreased by 56.1% over the 10-year period (Table), this trend abated in more recent years (Figure 2). Linear regression indicated average decreases of 259.1 cases per year (R^2^ = 0.92) from 2008 to 2013 and of 78.0 cases per year (R^2^, 0.93) from 2015 to 2017, confirming this observation. The number of cases decreased in most countries (e.g. Ireland, Germany and the UK; Supplementary Figure S2 shows the evolution of cases by serogroup and country); by contrast, cases remained relatively stable in some countries (e.g. Hungary, Latvia, Lithuania, and Poland) and increased in the Netherlands and Slovakia from 2014 and in Italy from 2015.

Black circles indicate incidence in individual countries and are associated with the left y-axis; red diamonds and red lines indicate the number of cases in Europe (26 countries included) and are associated with the right y-axis. Boxes indicate 25th, 50th and 75th percentiles of incidence rates for each group; whiskers indicate lowest and highest data point excluding any outliers.

**Figure 3:**
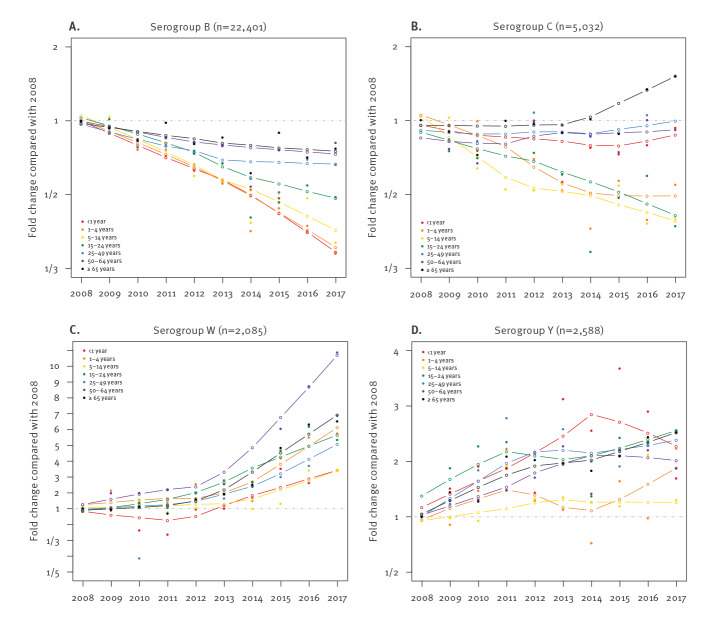
Evolution of invasive meningococcal disease incidence due to serogroups B, C, W and Y, by age group, Europe, 2008–2017 (n = 32,106)

The decreasing trend of serogroup B IMD during the period 2008 to 2017 in Europe was greater among younger age groups (64.3% reduction among infants) than older ones (27.4% reduction in individuals ≥ 65 years-old) as measured using curve fitting (Figure 3). The most recent trends (2015–2017) indicate that older age groups experienced increases in serogroup B incidence in more countries than younger age groups (Supplementary Figure S3 shows the change in incidence and number of cases between 2015 and 2017 by serogroup, age group and country). However, these increases are generally associated with a small number of additional cases because the incidence in these age groups is comparatively low. One-third (7/21) of countries also experienced recent increases in incidence rates among adolescents and young adults.

Evolution of incidence is expressed as fold changes in comparison with 2008. Dashed lines correspond to 1 (i.e. no change compared with 2008). Filled circles represent reported data, whereas hollow circles and corresponding lines represent smoothed data.

### Serogroup C

The incidence of serogroup C IMD in Europe was 0.10 per 100,000 in 2017 (Table); rates were highest in infants (1.04/100,000), followed by children 1–4 years-old (0.25/100,000), adolescents and young adults (0.14/100,000), and older adults (0.10/100,000; Figure 1). Proportions of cases were relatively homogenous among these age groups, with 21.4%, 15.5% and 20.4% of cases occurring among those 0–4, 15–24 and ≥ 65 years of age, respectively. Incidence varied among countries, with a substantial number of countries reporting no cases in various age groups (Figure 1).

The incidence of serogroup C IMD in Europe decreased by 30.4% over the 10-year period, from 0.14 to 0.10 per 100,000 (Table), despite temporary increases during two short periods (2010–2012 and 2014–2016; Figure 2). The proportion of IMD caused by serogroup C remained similar throughout the period 2008 to 2017, ranging from 13.0% to 15.7% (data not shown), and was only replaced in 2017 by serogroup W as the second most prevalent serogroup.

The greatest reductions in serogroup C incidence from 2008 to 2017 were observed in those 1–24 years of age (reductions of 46.7–58.9%; Figure 3). However this decline abated in those 1–4 years of age during the most recent years. By contrast, incidence in adults 65 years and older remained relatively constant through 2013, at which point it began to increase (increase of 86.0% since 2013); the increase in this age group was observed for the majority of individual countries in a comparison of 2017 with 2015 incidence that is provided in Supplementary Figure S3. Considering all age groups, some countries experienced reductions in cases (e.g. Germany, Austria, Poland), whereas others experienced increases over the study period (e.g. UK) or the more recent years of the study period (e.g. Czechia, France, Ireland, Italy, the Netherlands and Spain; provided as additional information in Supplementary Figure S2).

### Serogroup W

The incidence of serogroup W IMD in Europe in 2017 was 0.10 per 100,000 across all ages (Table). The highest rate was in infants (0.88/100,000), followed by children 1–4 years of age (0.26/100,000); however, adults 65 years and older represented the highest proportion of cases (32.1%, n = 163/508), with an associated incidence of 0.17 per 100,000 (Figure 1). There were 76 cases (15.0%) reported among adolescents and young adults, with an associated incidence of 0.14 per 100,000.

Serogroup W incidence increased by 517% from 2008 to 2017 (from 0.02 to 0.10/100,000), mainly driven by a limited but increasing number of countries and affecting all age groups (Table). Serogroup W represented only 1.7% of the total number of cases in 2008 but began to increase in 2012 (Figure 2); by 2017, it was the second most prevalent serogroup, accounting for 16.0% of IMD cases (Table; Supplementary Figure S1). Linear regression for the period 2011 to 2014 indicated an average increase of 31.4 cases per year (R^2^ = 0.92), mainly driven by the UK (Figure 2). The rate of increase escalated between 2015 and 2017 (average increase of 111.7 cases per year during 2014 to 2017; R^2^ = 0.97), reflecting increasing cases in multiple countries, dominated by the Netherlands, France, Spain and Germany (breakdown by country in Supplementary Figure S3). Despite the overall increasing trend between 2015 and 2017, incidence remained stable in a few countries (see additional data in Supplementary Figures S2 and S3).

The greatest increase of serogroup W cases occurred among those 50–64 years of age (987% increase; Figure 3). Younger age groups had smaller but substantial increases (245%, 477% and 240% increase among individuals < 1, 1–4 and 5–14 years of age, respectively). Adolescents and young adults, adults 25–49 years-old, and adults 65 years and older experienced respective increases of 432%, 587% and 549%. Despite limitations that were due to low case numbers when evaluated by country and age group, we observed similar trends when considering data from 2015 to 2017 across individual countries (Supplementary Figure S3).

### Serogroup Y

The incidence of serogroup Y IMD in Europe in 2017 across all ages was 0.07 per 100,000 (Table). The highest rates were in infants (0.20/100,000), adults 65 years and older (0.15/100,000) and adolescents and young adults (0.13/100,000). However, median incidence was highest in adolescents and young adults because only a few countries reported cases in younger age groups (Belgium, France, Germany, Italy, Portugal and the UK; Figure 1). Adults 65 years and older represented 41.6% of all serogroup Y cases, whereas 20.6% of cases occurred in adolescents and young adults. In 2017, eight countries (Belgium, Denmark, Finland, France, the Netherlands, Norway, Sweden and the UK) had notably higher incidences than other countries (Figure 2; Supplementary Figure S1).

Serogroup Y cases increased by 137.1% in Europe between 2008 and 2017, from 0.03 to 0.07 per 100,000; corresponding percentages among all IMD cases increased from 3.0% to 10.9% (Table). This increase in incidence was generally gradual except for notable increases in Norway and Sweden in earlier years (Figure 2; Supplementary Figure S2). Linear regression indicated an average increase of 19.22 cases per year across Europe (R^2^ = 0.81) from 2008 to 2017.

Analysing the evolution of serogroup Y incidence for individual age groups revealed increasing trends in all age groups (Figure 3). The rate of increase among infants was high until 2014, after which incidence began to decrease; more recent trends (2015–2017) among individual countries indicated that this decrease was mainly driven by France and the UK (Supplementary Figure S3). For older age groups (≥ 15 years), in which most cases occurred, rates of increase demonstrated less fluctuation (Figure 3). Adolescents and young adults experienced the highest increases during the 10-year period, with an increase of 156% (Figure 3).

### Other serogroups and non-groupable isolates

IMD caused by serogroups other than B, C, W and Y and non-groupable isolates represented a limited and stable proportion of cases compared with serogroups B, C, W and Y, considering the heterogeneity within this group (Table). There was a 32.8% decrease in incidence over the study period for this group, similar to the reduction observed for all IMD cases. No additional analyses were performed.

### Evolution of invasive meningococcal disease by age group

**Figure 4:**
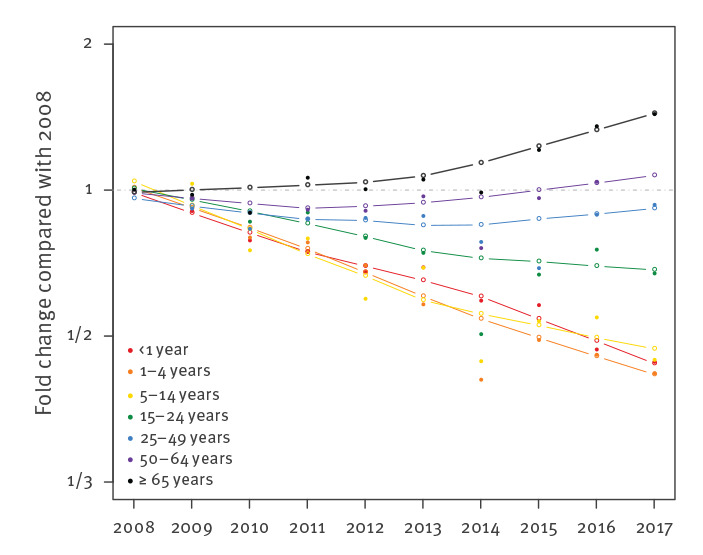
Evolution of invasive meningococcal disease incidence (all serogroups and non-groupable), by age group, Europe, 2008–2017 (n = 35,736)

Analysis of IMD incidence evolution (i.e. all serogroups and non-groupable) by age group from 2008 to 2017 highlighted very different trends among the different age groups (Figure 4). Infants and children 1–4 years-old demonstrated the greatest decreases in incidence, at 54.1% and 55.7%, respectively. Conversely, adults 65 years and older demonstrated a 51.7% increase in incidence. Intermediate age groups were ordered by age within these extremes. Curve fitting indicated that adults 65 years and older experienced a year-on-year increase in incidence, whereas those 50–64 and 25–49 years of age initially experienced decreasing trends that began to increase in 2012 and 2014, respectively. A decline in incidence was initially seen for those 15–24 years of age, which became less pronounced from 2013 to 2014. A similar trend was observed for the 5–14-year-old group.

Data include all cases with identifiable serogroups as well as non-groupable isolates. Evolution of incidence is expressed as fold changes in comparison with 2008. The dashed line corresponds to 1 (i.e. no change compared with 2008). Filled circles represent reported data, whereas hollow circles and corresponding lines represent smoothed data.

## Discussion

Evaluating IMD epidemiology in Europe over a 10-year period enabled identification of clear trends despite the overall unpredictability of meningococcal disease. Although IMD incidence decreased by 34.4% from 2008 to 2017, there were dynamic changes in evolution among serogroups, countries and age groups. These primarily included the increasing disease incidence across all ages associated with serogroups W and Y and in the older population when considering all serogroups.

Serogroup B remained the most prevalent disease-causing serogroup in Europe in 2017, mainly affecting infants and young children but also adolescents and young adults. The 56.1% decline in serogroup B incidence over the 10-year period was the main driver of the overall decrease in IMD and the reason for this is unclear. During the study period, the introduction of smoking bans in public places in some countries [30] and changes in behaviours associated with bacterial transmission or infection, such as intimate kissing and nightclub attendance, have also been postulated as possible explanations [31,32], although the evidence of these factors being contributory is limited and speculative. However, the decline in serogroup B disease was not concurrently observed for other serogroups such as W and Y; it has therefore been suggested that factors contributing to the decrease in serogroup B disease may be specific to the genetic diversity of serogroup B or its competing organisms [31,33]. Interestingly, the rate of decrease has slowed and was mainly due to stabilisation of rates in adults 25 years and older. Continuation of this trend needs confirmation and the reasons clarified. The increasing incorporation of MenB vaccines into paediatric immunisation schedules as seen in Austria, Ireland, Italy, Lithuania and the UK [17] may partly explain some of the most recent data. However, these vaccines were only introduced from late 2015 onward [25], whereas the trend of declining incidence predates implementation, was observed across all age groups, including those not targeted for vaccination, and was also observed in countries that did not implement MenB vaccination programmes.

In spite of low incidence, there was a rapid and marked increase in serogroup W disease in Europe and other regions including Latin America and Australia [11-14,34,35]. In Europe, the initial increase was mainly restricted to the UK and caused by the so-called 'original UK strain', a hyperinvasive cc11 strain originating from Latin America [36]. This strain subsequently mutated to a strain named 'UK 2013 strain' and spread to other European countries [14]. This resulted in several countries, including Belgium, the Netherlands, Spain, Switzerland and the UK, implementing MenACWY vaccination programmes [14,19-23,37]. National MenACWY recommendations have predominantly targeted adolescents to provide both direct and indirect protection [14,19-24,37]. Because of their recent implementation, there is little evidence of impact from these programmes on serogroup W cases in the presented data, although 2017 data for the UK, one of the first European countries to implement MenACWY vaccines in a national programme (in August 2015 [37]), showed an initial decrease in serogroup W IMD compared with 2016. These vaccination programmes are expected to considerably impact future epidemiology of serogroup W as well as serogroup Y, which has been increasing in Europe. This is supported by post-2017 data from England showing both direct and indirect impact of MenACWY vaccination on these serogroups [37,38].

Vaccination programmes for serogroup C have been highly effective, leading to the reduction in cases in Europe that largely occurred in the years preceding the period included in this analysis [8,18]. However, some countries with well-established MenC vaccination programmes, such as the UK and Ireland [8,18,39], experienced increases in serogroup C case numbers across multiple age groups during the study period, although incidence is still low. These increases highlight the importance of maintaining robust population immunity, even when case numbers are low, and also suggest that the optimal approach for disease control may rely on both direct and indirect protection of at-risk age groups.

One of the most important findings of the current analysis is the age-related evolution of IMD incidence in Europe. Depending on serogroup, older age groups have experienced either the lowest decline (serogroup B) or the highest increases (serogroups C, W and Y). In addition, older individuals experienced the earliest rise in serogroup W IMD associated with cc11 clonal expansion [14]. It is possible that the growing population of older adults [40], the diversification of their social activities and the evolution of intergenerational relationships may increase their interaction rates, calling into question the previously assumed minor role of this age group in transmission [24]. Other hypotheses, such as variations in individual immunity, the emergence of clones mainly affecting adults, or the emergence of low virulent strains likely acquired from other age groups, should also be considered. It is not clear why some serogroups or clones are associated with specific age groups and do not evolve in the same manner. Increasing incidence of IMD caused by serogroups commonly associated with older age groups (e.g. serogroup Y) may be a cause or an effect of the general increases in IMD observed among older adults. However, the slowing decrease of serogroup B among older adults supports a general increase of IMD in this age group. This evolution may indicate an increasing contribution of adults to the burden of IMD in the future.

Adolescents and young adults are presumed to be the main transmitters of IMD [24]. Also, this age group experienced a substantial increase in IMD caused by serogroups W and Y between 2008 and 2017, and a more limited decrease in serogroup B disease compared with younger age groups. These observations support the hypothesis that this age group may continue to contribute to the burden of IMD in the future, unless vaccination programmes are implemented in this age group. When traveling, adolescents and young adults are exposed to the disease epidemiology of other countries and may disseminate the pathogen internationally. This possibility was exemplified by a cluster of six serogroup W IMD cases in Scotland and Sweden that were linked to individuals returning from the 2015 World Scout Jamboree in Japan [41].

A major limitation of the current analysis is the difficulty in precisely assessing the effect of existing vaccination programmes on the epidemiology of IMD. Because this effect is influenced by the characteristics of the vaccination programme, including the approach to implementation, vaccines used, recommended schedules and uptake rates, in-depth country-specific analyses are required. Another major limitation of this study is the variability and evolution in the quality of the surveillance systems in individual countries and data completeness, thereby affecting the comparability of the data between countries.

## Conclusion

This review of European IMD epidemiology from 2008 to 2017 reconfirms that IMD can evolve rapidly both in terms of the clones and serogroups causing disease as well as the age groups affected. The rapid expansion of cc11 serogroup W is well reported; however, the trend analysis reported here also highlights an increasing incidence of serogroup Y IMD across all age groups and all-serogroup IMD in older age groups in spite of low incidence at the start of the study period. Epidemiological monitoring is essential for evaluating vaccination implementation strategies in terms of identifying disease epidemiology as well as assessing the impact of incumbent vaccination programmes. We have recently entered an era distinguished, for the first time, by availability of vaccines for the prevention of the most prevalent serogroups. The recent replacement of MenC vaccines with MenACWY vaccines is an example of how some countries have responded to changing epidemiological trends. The increasingly widespread use of MenB and MenACWY vaccines throughout European countries has the potential to positively impact IMD epidemiology by reducing the current disease burden as well as preventing increases in cases associated with outbreaks and emergence of clones or serogroups not covered by the current vaccination programmes.

## Supplementary Data

920000 Supplement
