# Supplementary material for: Evolution of invasive meningococcal disease epidemiology in Europe, 2008 to 2017
Source: Euro Surveill. 2022 Jan 20;27(3):2002075. doi: 10.2807/1560-7917.ES.2022.27.3.2002075 (PMC8804660; doi:10.2807/1560-7917.ES.2022.27.3.2002075)
Supplement: Supplement [file 20-02075_NUTTENS_Supplement.pdf]

Supplementary Material

This supplementary material is hosted by *Eurosurveillance* as supporting information alongside the article “Evolution of Invasive Meningococcal Disease Epidemiology in Europe” on behalf of the authors, who remain responsible for the accuracy and appropriateness of the content. The same standards for ethics, copyright, attributions, and permissions as for the article apply. *Eurosurveillance* is not responsible for the maintenance of any links or email addresses provided therein.

Supplemental Figure 1. Incidence of IMD due to serogroups B, C, W, and Y for individual countries and Europe, 2008 and 2017.

Data are for individual countries (black circles) and Europe (26 countries included; red diamonds). Boxes indicate 25th, 50th, and 75th percentiles of incidence rates for each group; whiskers indicate lowest and largest data point excluding any outliers. CZE=Czech Republic; FRA=France; GBR=United Kingdom; IRL=Ireland; LTU=Lithuania; NLD=Netherlands.

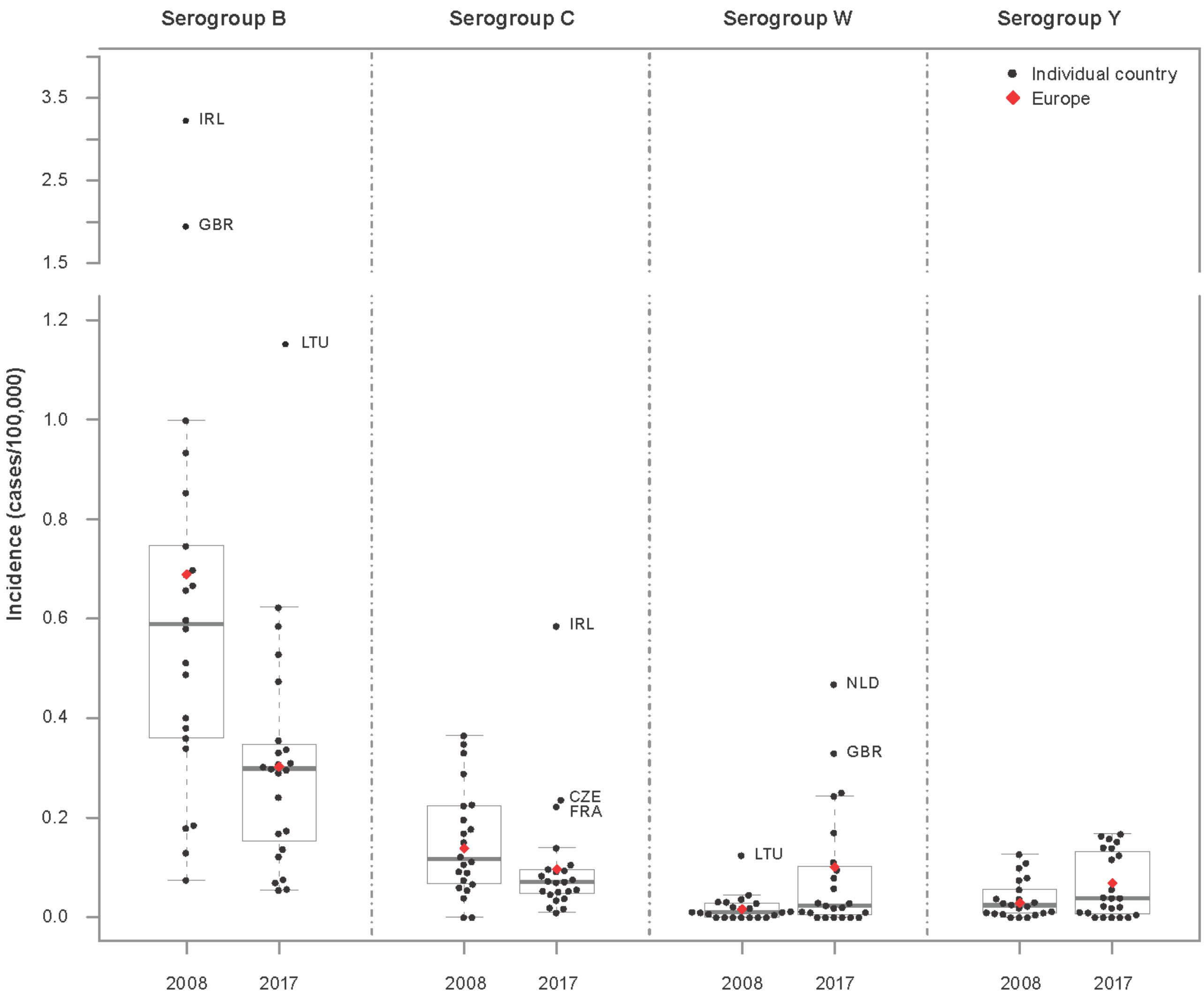

Supplemental Figure 2. Evolution of the number of IMD cases due to serogroups B, C, W, and Y for individual countries, 2008–2017.

A. Serogroup B

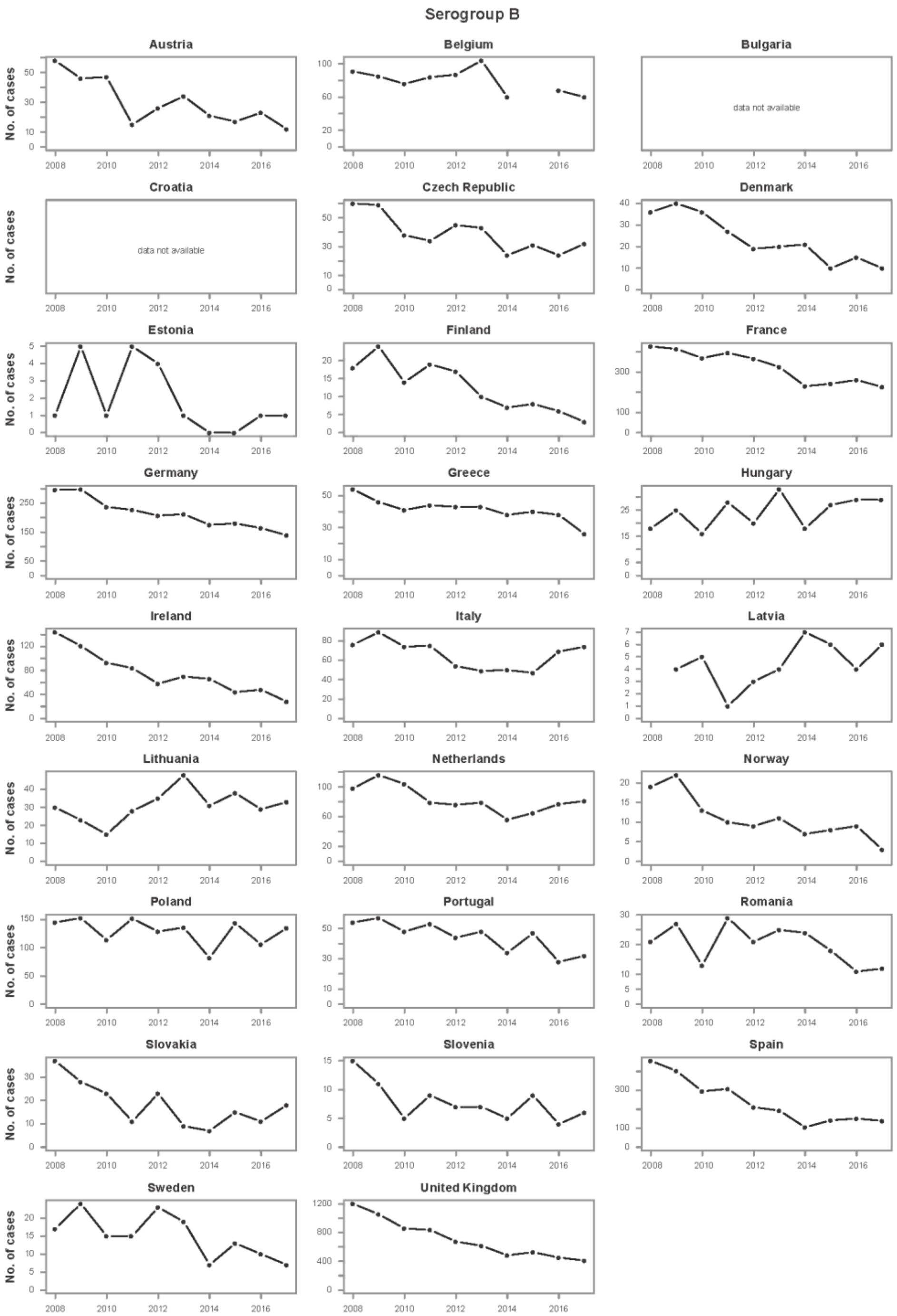

B. Serogroup C

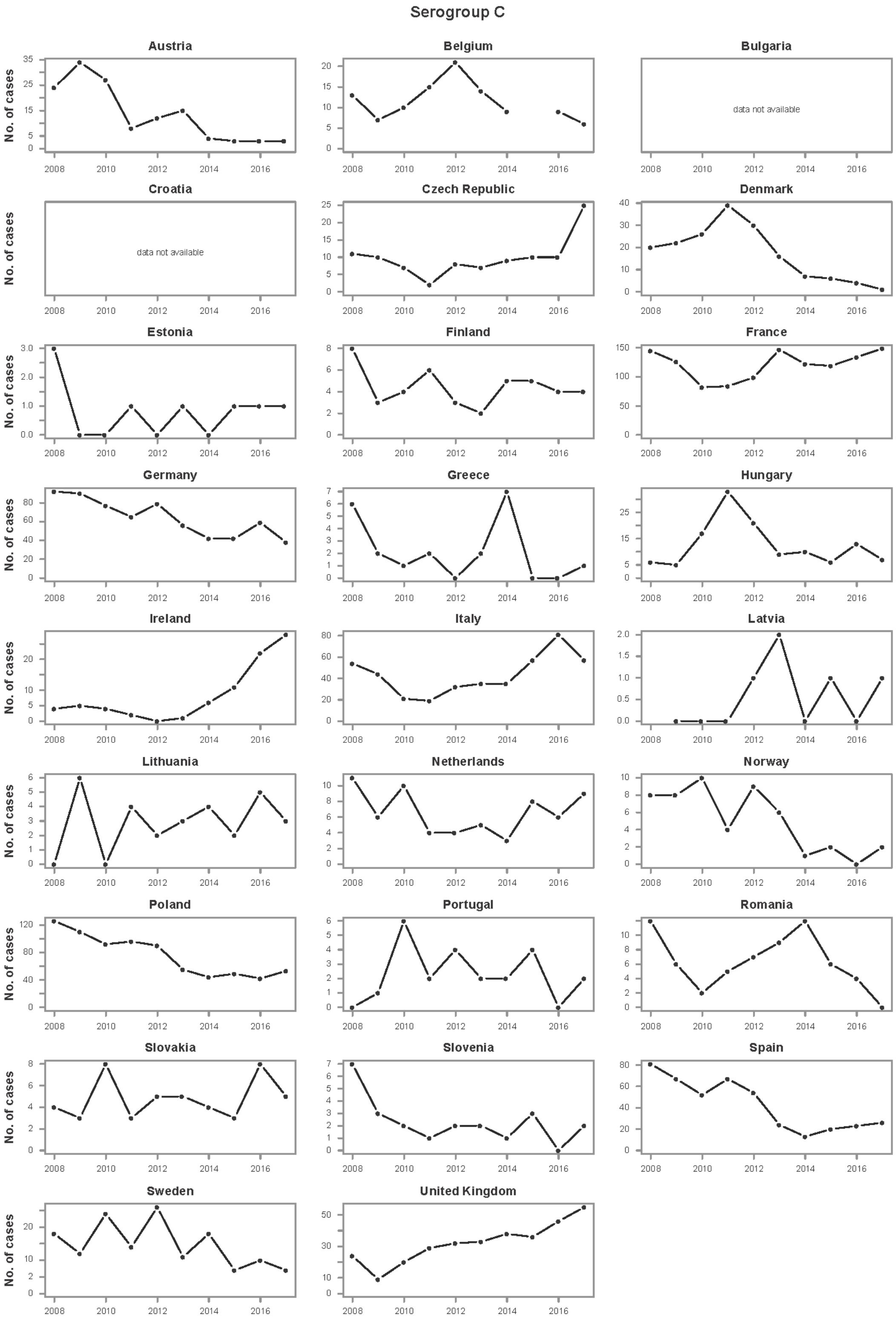

C. Serogroup W

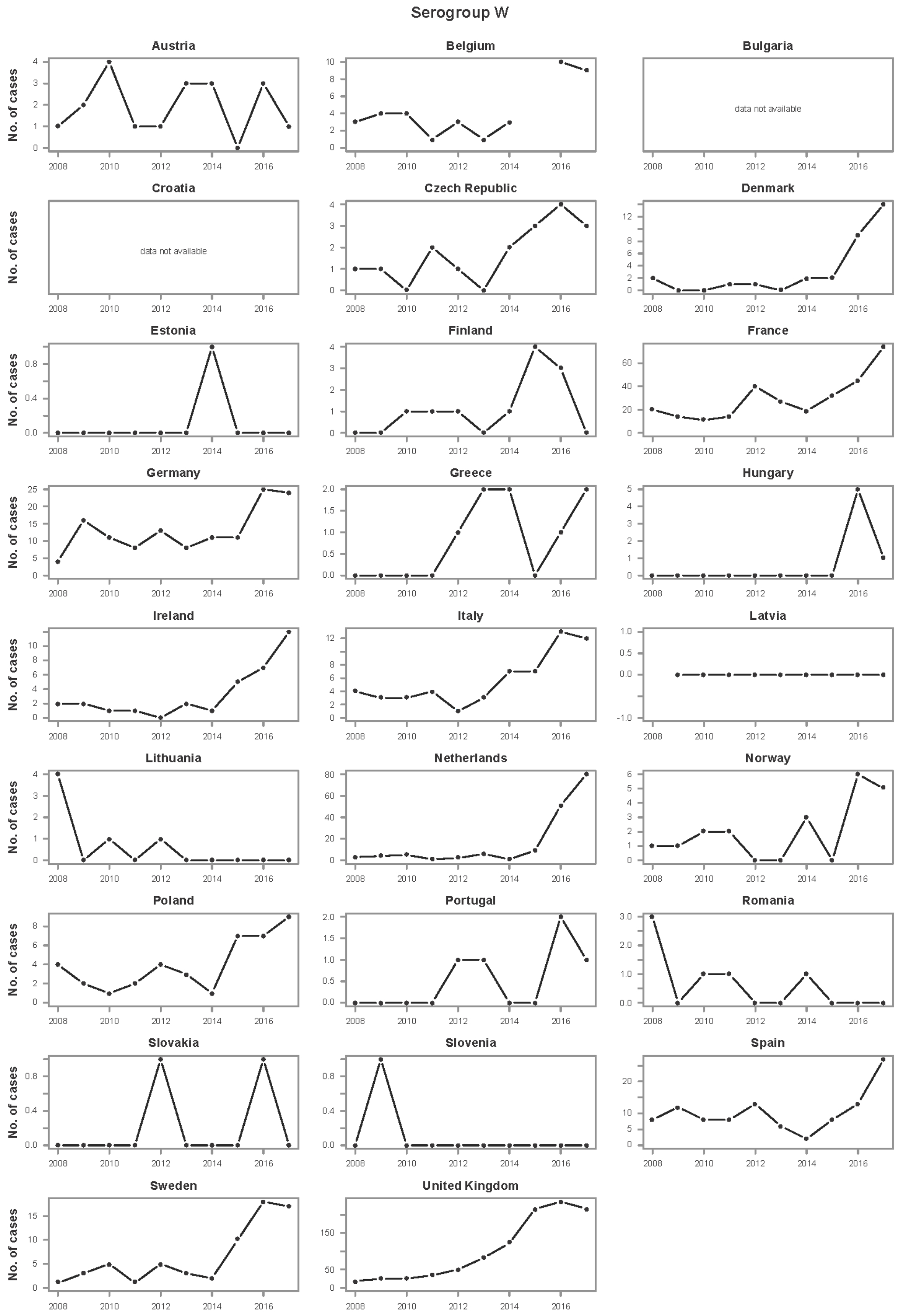

D. Serogroup Y

Serogroup Y

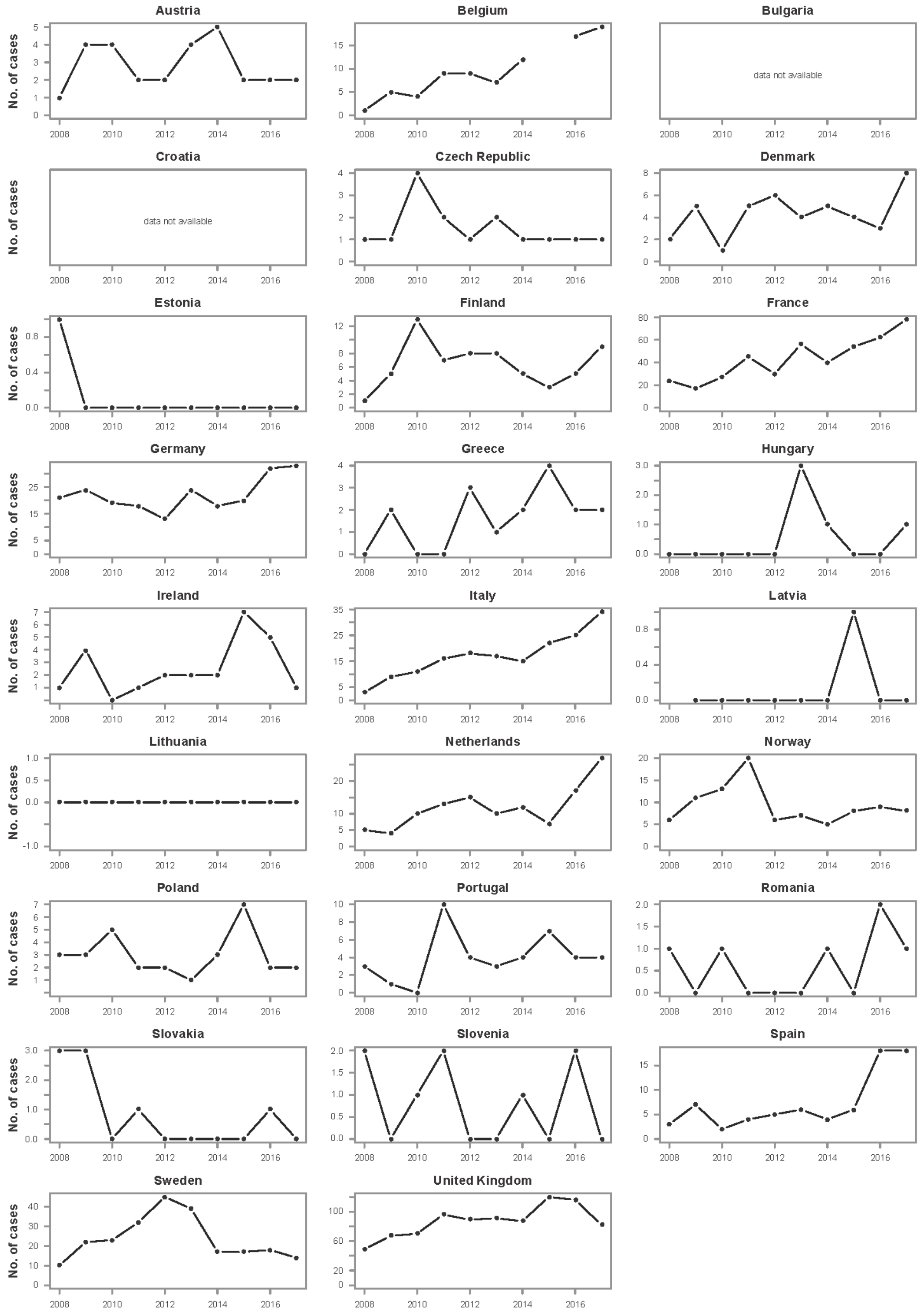
Austria
Belgium
Bulgaria
data not available
Croatia
data not available
Czech Republic
Denmark
Estonia
Finland
France
Germany
Greece
Hungary
Ireland
Italy
Latvia
Lithuania
Netherlands
Norway
Poland
Portugal
Romania
Slovakia
Slovenia
Spain
Sweden
United Kingdom
No. of cases
2008
2010
2012
2014
2016

Supplemental Figure 3. Two-year evolution of IMD incidence due to serogroups B, C, W, and Y occurring in individual countries, 2017 vs 2015.

Evolution of incidence is expressed as fold changes comparing 2017 to 2015 for all age groups combined (labeled as “all”) and by individual age groups. Countries having no cases in 2015 and 1 or more cases in 2017 will result in a fold change of +∞ and are shown using the darkest red (ie, 4 and above). Similarly, countries having 1 or more cases in 2015 and no cases in 2017 will result in a fold change of 0 and are shown using the darkest blue (ie, 1/4 and below). An incidence of 0 in specific age groups is likely related to the population sizes of the individual countries. Countries with missing incidence data for either 2017, 2015 (ie, Belgium), or both are colored gray. The absolute difference in number of cases between 2015 and 2017 is indicated as a numeric value within each cell.

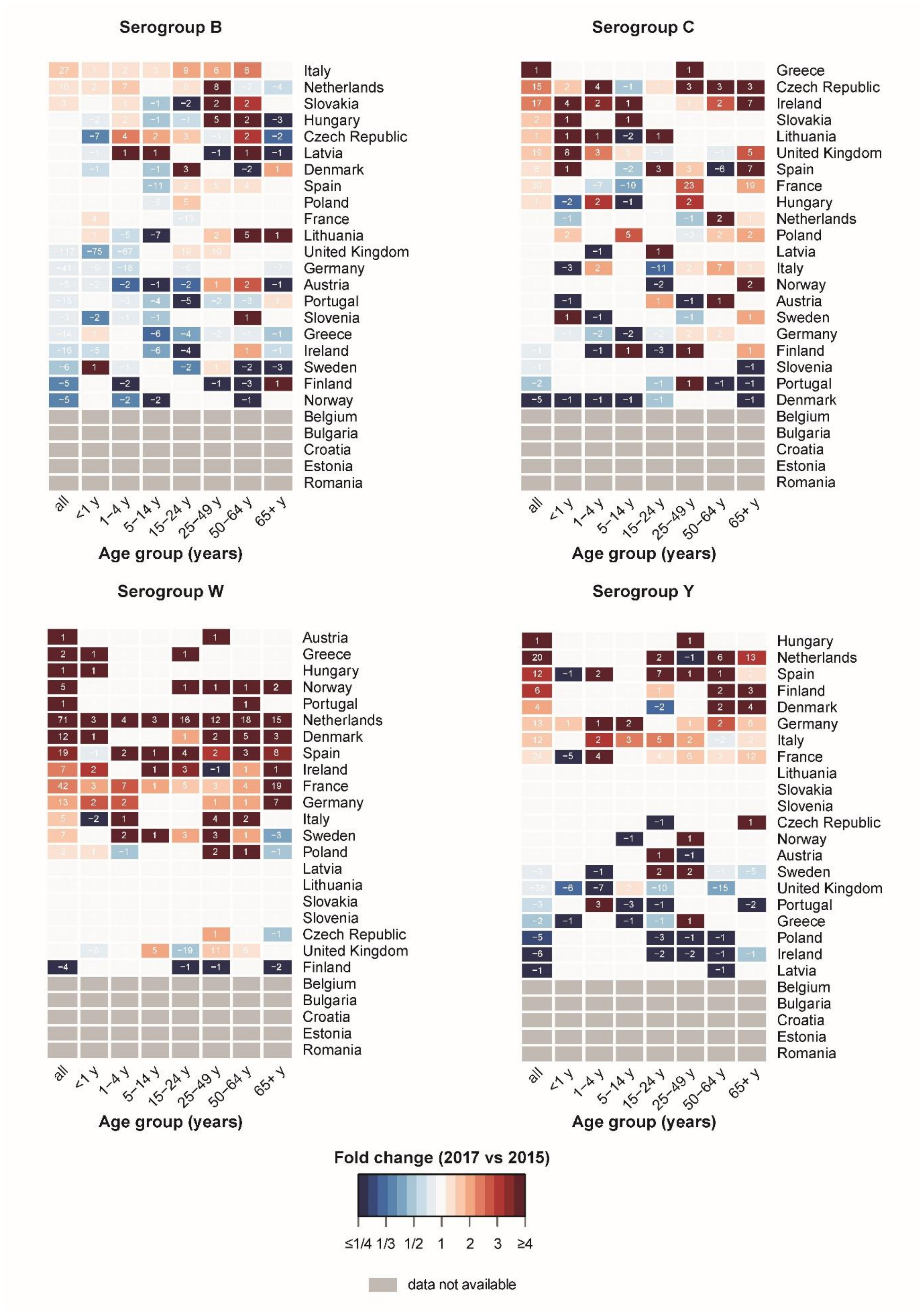
Serogroup B
Italy
Netherlands
Slovakia
Hungary
Czech Republic
Latvia
Denmark
Spain
Poland
France
Lithuania
United Kingdom
Germany
Austria
Portugal
Slovenia
Greece
Ireland
Sweden
Finland
Norway
Belgium
Bulgaria
Croatia
Estonia
Romania
all
<1 y
1–4 y
5–14 y
15–24 y
25–49 y
50–64 y
65+ y
Age group (years)
Serogroup C
Greece
Czech Republic
Ireland
Slovakia
Lithuania
United Kingdom
Spain
France
Hungary
Netherlands
Poland
Latvia
Italy
Norway
Austria
Sweden
Germany
Finland
Slovenia
Portugal
Denmark
Belgium
Bulgaria
Croatia
Estonia
Romania
Age group (years)
Serogroup W
Austria
Greece
Hungary
Norway
Portugal
Netherlands
Denmark
Spain
Ireland
France
Germany
Italy
Sweden
Poland
Latvia
Lithuania
Slovakia
Slovenia
Czech Republic
United Kingdom
Finland
Belgium
Bulgaria
Croatia
Estonia
Romania
Age group (years)
Serogroup Y
Hungary
Netherlands
Spain
Finland
Denmark
Germany
Italy
France
Lithuania
Slovakia
Slovenia
Czech Republic
Norway
Austria
Sweden
United Kingdom
Portugal
Greece
Poland
Ireland
Latvia
Belgium
Bulgaria
Croatia
Estonia
Romania
Age group (years)
Fold change (2017 vs 2015)
≤1/4 1/3 1/2 1 2 3 ≥4
data not available
